# Polymorphisms within Immune Regulatory Pathways Predict Cetuximab Efficacy and Survival in Metastatic Colorectal Cancer Patients

**DOI:** 10.3390/cancers12102947

**Published:** 2020-10-13

**Authors:** Nico B. Volz, Diana L. Hanna, Sebastian Stintzing, Wu Zhang, Dongyun Yang, Shu Cao, Yan Ning, Satoshi Matsusaka, Yu Sunakawa, Martin D. Berger, Chiara Cremolini, Fotios Loupakis, Alfredo Falcone, Heinz-Josef Lenz

**Affiliations:** 1 Division of Medical Oncology, Norris Comprehensive Cancer Center, University of Southern California, Los Angeles, CA 90033, USA; nico.volz@mountsinai.org (N.B.V.); diana.hanna@med.usc.edu (D.L.H.); sebastian.stintzing@charite.de (S.S.); Wu.Zhang@med.usc.edu (W.Z.); Yan.Ning@med.usc.edu (Y.N.); matsusaka-s@md.tsukuba.ac.jp (S.M.); y.sunakawa@marianna-u.ac.jp (Y.S.); martin.berger@insel.ch (M.D.B.); 2 Department of Emergency Medicine, Icahn School of Medicine at Mount Sinai, New York, NY 10029, USA; 3 Department of Medicine III, University Hospital LMU Munich, 80539 Munich, Germany; 4 Department of Preventive Medicine, Norris Comprehensive Cancer Center, University of Southern California, Los Angeles, CA 90033, USA; donyang@coh.org (D.Y.); shucao@stanford.edu (S.C.); 5 U.O. Oncologia Medica 2—Aziendo Ospedaliero-Universitaria Pisana, 56126 Pisa, Italy; chiara.cremolini@unipi.it (C.C.); fotios.loupakis@iov.veneto.it (F.L.); alfredo.falcone@med.unipi.it (A.F.)

**Keywords:** CD24, biomarker, cetuximab, colorectal cancer, single nucleotide polymorphism

## Abstract

**Simple Summary:**

Cetuximab is an antibody that blocks EGFR signaling and stimulates an immune response against cancer cells. For patients with advanced colorectal cancer, tumor sidedness and *RAS* mutation status are the primary factors used to select systemic therapy. Additional biomarkers are needed to better predict which patients will benefit from cetuximab-based regimens. The aim of our retrospective study was to assess the predictive and prognostic value of 12 germline single nucleotide polymorphisms in five immune related genes in 924 patients with advanced colorectal cancer undergoing therapy with cetuximab. We identified a *CD24* germline genetic variant which independently predicted survival in a discovery cohort and confirmed these findings in a validation cohort. If confirmed in prospective studies, *CD24* and other immune related polymorphisms may guide the use of cetuximab in patients with advanced colorectal cancer.

**Abstract:**

Cetuximab, an IgG1 EGFR-directed antibody, promotes antibody-dependent cell-mediated cytotoxicity. We hypothesized that single-nucleotide polymorphisms (SNPs) in immune regulatory pathways may predict outcomes in patients with metastatic colorectal cancer treated with cetuximab-based regimens. A total of 924 patients were included: 105 received cetuximab in IMCL-0144 and cetuximab/irinotecan in GONO-ASL608LIOM01 (training cohort), 225 FOLFIRI/cetuximab in FIRE-3 (validation cohort 1), 74 oxaliplatin/cetuximab regimens in JACCRO CC-05/06 (validation cohort 2), and 520 FOLFIRI/bevacizumab in FIRE-3 and TRIBE (control cohorts). Twelve SNPs in five genes (*IDO1*; *PD-L1*; *PD-1*; *CTLA-4*; *CD24*) were evaluated by PCR-based direct sequencing. We analyzed associations between genotype and clinical outcomes. In the training cohort; patients with the *CD24* rs52812045 A/A genotype had a significantly shorter median PFS and OS than those with the G/G genotype (PFS 1.3 vs. 3.6 months; OS 2.3 vs. 7.8 months) in univariate (PFS HR 3.62; *p* = 0.001; OS HR 3.27; *p* = 0.0004) and multivariate (PFS HR 3.18; *p* = 0.009; OS HR 4.93; *p* = 0.001) analyses. Similarly; any A allele carriers in the JACCRO validation cohort had a significantly shorter PFS than G/G carriers (9.2 vs. 11.8 months; univariate HR 1.90; *p* = 0.011; multivariate HR 2.12; *p* = 0.018). These associations were not demonstrated in the control cohorts. *CD24* genetic variants may help select patients with metastatic colorectal cancer most likely to benefit from cetuximab-based therapy.

## 1. Introduction

Cetuximab monotherapy and combinations with FOLFIRI (leucovorin, fluorouracil, irinotecan) or FOLFOX (leucovorin, fluorouracil, oxaliplatin) have prolonged the survival of metastatic colorectal cancer (mCRC) patients with *RAS* wild-type tumors [1,2]. Nonetheless, one-third of *RAS*-selected patients do not benefit from cetuximab-based regimens [3], and treatment resistance develops independent of RAS/RAF/MEK signaling.

Cetuximab, a chimeric IgG1 monoclonal antibody against the epidermal growth factor receptor (EGFR), promotes antibody-dependent cell-mediated cytotoxicity (ADCC) [4,5]. Upon binding to the FCγ receptor on natural killer (NK) and other immune cells, cetuximab facilitates Fas-FasL-induced tumor cell apoptosis. In response to cetuximab-mediated ADCC, compensatory immunosuppressive pathways (e.g., PDL-1, CTLA-4) may be activated by cancer cells [6]. While extended *RAS* testing accounts for compensatory pathways that renders tumors resistant to cetuximab-mediated EGFR inhibition [3], there are no validated markers to predict the benefit from its ADCC-dependent mode of action. Identifying such markers could refine patient selection and reveal novel actionable alterations.

We previously demonstrated the prognostic value of FCγ-receptor polymorphisms in mCRC patients receiving cetuximab [7]. With the advent of immune checkpoint inhibitors in mCRC, identifying relevant biomarkers is an area of active investigation. We hypothesized that single nucleotide polymorphisms (SNPs) within critical immune regulatory pathways may serve as clinically meaningful markers in mCRC patients receiving cetuximab-based therapy. We examined 12 SNPs in five genes (*CD24*, *IDO1*, *PD-L1*, *PD-1*, *CTLA-4*) to identify predictive and prognostic genetic variants in a training cohort of mCRC patients treated with cetuximab with or without irinotecan in two phase 2 studies [8]. Then, we tested SNPs with significant prognostic associations in two validation cohorts of mCRC patients, one treated with FOLFIRI-cetuximab in FIRE-3 [2] and the other treated with oxaliplatin-cetuximab regimens in JACCRO CC-05/06. Lastly, we examined these associations in two control cohorts who received FOLFIRI-bevacizumab in FIRE-3 and TRIBE [2,9].

## 2. Results

Patient baseline characteristics and primary outcome data are summarized in Table 1. Patients in the training cohort had relatively refractory disease, with lower overall response rate (RR) (20.0%; *p* < 0.001) and disease control rate (61.9%; *p* < 0.001), and an expectedly shorter median PFS (3.7 months, 95% CI: 3.2, 4.9 months; *p* < 0.001) and OS (10.6 months, 95% CI: 7.8, 13.2 months; *p* < 0.001) than those in the validation or control cohorts who underwent first-line therapy.

### 2.1. Gene Variants and OS

Within the training cohort, three polymorphisms (*CD24* rs52812045, *IDO1* rs3739319, *IDO1* rs9657182) were significantly associated with OS. 

**Figure 1:**
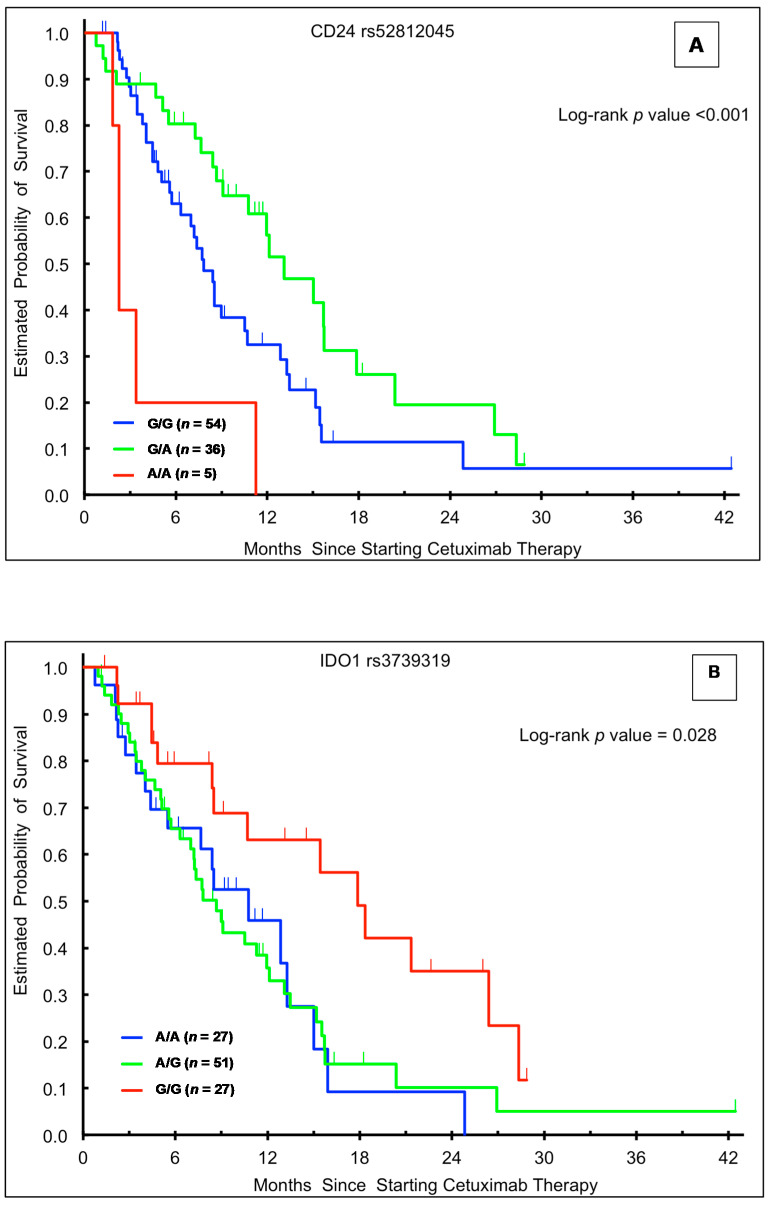

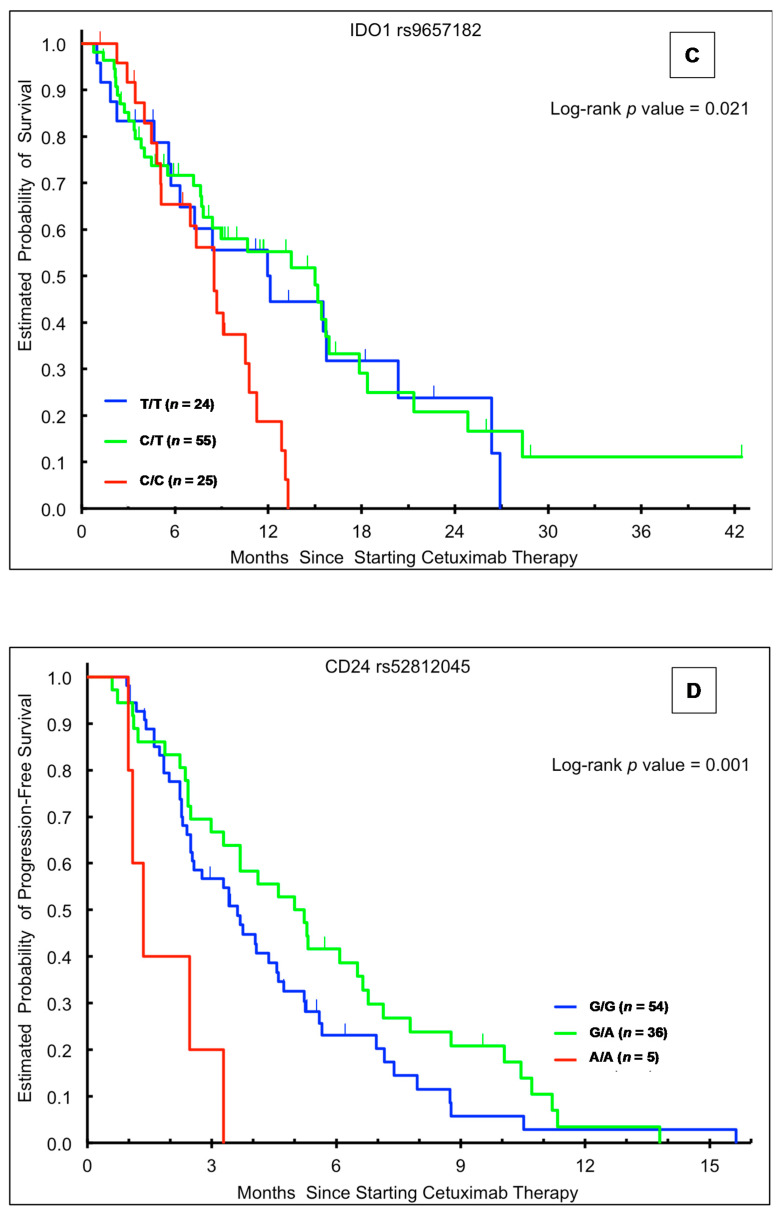
Kaplan-Meier plots according to genotype and outcome in the training cohort. (**A**) Overall survival (OS) stratified by *CD24* rs52812045 genotype; (**B**) OS stratified by *IDO1* rs3739319 genotype; (**C**) OS stratified by *IDO1* rs9657182 genotype; (**D**) progression-free survival stratified by *CD24* rs52812045 genotype.

In the univariable analysis, patients in the training cohort with the *CD24* rs52812045 G/A genotype had a superior median OS relative to those with either homozygous genotype (G/A 13.1 vs. G/G 7.8 vs. A/A 2.3 months, HR 0.58, *p* < 0.001; Table 2, Figure 1A). This association held significance in a multivariable model accounting for sex, age, ethnicity, and presence of rash (HR 0.50, 95% CI: 0.28–0.88, *p* = 0.001; Table 2). There were no significant associations between *CD24* rs52812045 and OS in the validation (Table 3 and Table 4) or control (Table 5 and Table 6) cohorts.

Patients in the training cohort carrying the *IDO1* rs3739319 G/G variant had a significantly longer median OS (17.9 months) compared to those with the A/A (10.8 months, HR 2.13, 95% CI: 1.04–4.36) or A/G (8.7 months, HR 2.12, 95% CI: 1.15–3.93) genotypes (*p* = 0.028; Table 2, Figure 1B). This association held significance in multivariable analysis (*p* = 0.043). There were no significant associations between *IDO1* rs3739319 genotype and OS in the other cohorts. In the FIRE-3 validation cohort, patients with an *IDO1* rs3739319 A allele had a numerically longer OS (33.1 months) in univariate (HR 0.72, 95% CI: 0.50–1.03, *p* = 0.068) and multivariate (HR 0.70, 95% CI: 0.48–1.02, *p* = 0.063) analyses, though this association did not reach statistical significance (Table 3). 

Patients in the training cohort with the *IDO1* rs9657182 C/T (15.0 months, HR 0.49, 95% CI: 0.27–0.89) genotype had a significantly longer OS than those harboring the C/C genotype (8.5 months) (*p* = 0.021; Table 2, Figure 1C). This relationship remained significant in multivariable analysis (*p* = 0.008; Table 2). Conversely, in the Japanese validation cohort, patients with the heterozygote C/T genotype had the shortest median OS (26.5 vs. 41.1+ months for C/C genotype; univariate HR 2.18, 95% CI 0.98–4.85), though with borderline significance (*p* = 0.049). There was a similar but non-significant trend in multivariate analysis (*p* = 0.12) (Table 4). *IDO1* rs9657182 genotype was not significantly associated with OS in the other cohorts (Table 3,Table 5 and Table 6). 

Patients in the TRIBE control cohort with any *PD-L1* rs2297137 A allele had a significantly prolonged OS (33.9 months) compared to those with the G/G genotype (23.4 months) in univariable (HR 0.63, 95% CI 0.45-0.89, *p* = 0.007) and multivariable analyses (HR 0.65, 95% CI 0.45–0.94, *p* = 0.021) (Table S6). This association was not seen in the other cohorts.

### 2.2. Gene Variants and PFS

One polymorphism (*CD24* rs52812045) was significantly associated with PFS in the training cohort. 

In the training cohort, the *CD24* rs52812045 A/A genotype was associated with shorter median PFS (1.3 months) in both univariable (HR 3.62, 95% CI: 1.35–9.70, *p* = 0.001) and multivariable analyses (HR 3.18, 95% CI: 1.10–9.20, *p* = 0.009) (Table 2, Figure 1D). Similarly, in the Japanese validation cohort, the A allele was associated with significantly shorter PFS in univariable (HR 1.90, 95% CI: 1.05–3.42, *p* = 0.011) and multivariable (HR 2.12, 95% CI: 1.14–3.95, *p* = 0.018) analyses (Table 4). There were no significant associations between *CD24* rs52812045 and PFS in the other cohorts (Table 3,Table 5 and Table 6).

In the FIRE-3 control cohort, patients harboring a T allele of *IDO1* rs9657182 had a significantly shorter PFS (9.8 months) compared to those with the C/C genotype (13.2 months) in univariable (HR 1.45, 95% CI: 1.08–1.95, *p* = 0.010) and multivariable analyses (HR 1.44, 95% CI: 1.06–1.96, *p* = 0.019) (Table 5). This association was not demonstrated in the other cohorts.

Patients in the FIRE-3 validation cohort with any A allele of *IDO1* rs3739319 achieved a prolonged PFS compared to those with the G/G genotype, but only in multivariable analysis (10.5 vs. 9.2 months; univariable HR 0.76, 95% CI: 0.57–1.02, *p* = 0.059; multivariable HR 0.67, 95% CI: 0.50–0.91, *p* = 0.011) (Table 3). This trend was not evident in the other cohorts.

Patients in the training cohort with the *CTLA4* rs231777 C/T genotype had a significantly shorter PFS (2.6 months) compared to those with the C/C variant (4.1 months), but only in multivariable analysis (HR 1.76, 95% CI: 1.10–2.81, *p* = 0.019) (Table S2). This association was not seen in the other cohorts.

### 2.3. Gene Variants and Tumor Response

In the training cohort, patients with the *PD-L1* rs2297137 A/A genotype demonstrated a greater RR (56%) than patients with G/A (19%) or G/G (16%) genotypes (two-sided Fisher’s exact test *p* = 0.029) (Table S2). There was no association between *PD-L1* genotype and RR in the validation or control cohorts.

## 3. Discussion

Cetuximab affects EGFR inhibition as well as innate and adaptive immunity [10,11,12,13]. While *RAS* mutation status is a validated predictive marker for cetuximab in mCRC patients, it may not completely account for its immunomodulatory effects. 

The wide-ranging immunogenic effects of cetuximab have been demonstrated in multiple studies [6,10]. Cetuximab binds the Fc portion of the activating receptor on NK cells, which may lead to direct cancer cell lysis and the release of tumor antigens which can be presented by dendritic cells to cytotoxic T-cells [6]. Moreover, activated NK cells may use cytokines and interferon to activate tumor suppressing macrophages [6]. In CRC cell lines and murine xenograft models, cetuximab has been shown to induce immunogenic cell death, marked by increased phagocytosis by dendritic cells [10], as well as stimulating and enhancing NK cell activity [14]. The cytotoxic agents, 5-FU and irinotecan, have also been shown to improve the ADCC activity of cetuximab across different CRC cell lines, in part by increasing cell surface EGFR expression [15]. The interaction between *KRAS* mutations and the ability of cetuximab to mediate ADCC is not completely understood. Some studies have shown ADCC to be dependent on *KRAS*-wildtype status [16], while others show the effects to be independent of *KRAS* status [6]. In mCRC patients with liver-limited metastases undergoing pre-operative chemotherapy with or without cetuximab, cetuximab was shown to increase the degree of tumor infiltration with CD3+ and CD8+ T-cells in addition to CD56+ NK cells [11]. Clinically, the degree of ADCC activity (as measured in peripheral blood mononuclear cells and by NK-dependent LDH release) has been positively associated with improved survival [17], as well as degree of tumor shrinkage [18] in mCRC patients receiving cetuximab. 

Much of the literature regarding the predictive and prognostic impact of immune-related germline polymorphisms on cetuximab efficacy has focused on the FCγ-receptor family [7,19,20,21]. As immunotherapeutics become integrated into the management of advanced CRC, discovering markers of efficacy within immune mediated pathways will become increasingly important. To our knowledge, this is the first study to evaluate the predictive and prognostic utility of common genetic variants within checkpoint and other immune regulatory pathways in mCRC patients receiving cetuximab-based therapy. 

CD24, a membrane surface glycosyl-phosphatidyl-inositol-(GPI)-anchored protein, is overexpressed in 90% of colorectal tumors [22] and has been implicated in inflammatory bowel disease (IBD) [23,24]. As a putative marker of EGF and Wnt-expressing metaplastic Paneth cells [25,26], CD24 provides a crucial link between host mucosal immunity, colonic crypt homeostasis [27], and the intestinal stem cell niche [28,29]. Indeed, *CD24* knockout mice models exhibit reduced T-cell proliferation [30], blunted polyp formation, and suppressed colorectal tumorigenesis [22,31]. Furthermore, the effects of CD24 on ADCC and chronic inflammation are thought to be partly mediated by autophagy [27,32], another purported mechanism of cetuximab efficacy [33]. 

*CD24* maps to chromosome 6q21 and rs52812045 is located in the GPI-anchor cleavage site [34]. Clinically, this polymorphism has been associated with pathologic complete response and concomitant intra-tumoral lymphocyte infiltration in breast cancer patients [35,36], and poor prognosis in esophageal cancer patients [37]. However, a recent meta-analysis of *CD24* SNPs and cancer risk was non-confirmatory [38]. In our study, carriers of the *CD24* rs52812045 A/A variant in the training cohort had the shortest PFS and OS compared to those with the G/G and G/A genotypes, with heterozygotes appearing to have an OS advantage (albeit with the 95% CI boundary near 1 in univariable analysis). Similarly, in the Japanese validation cohort, the A allele was associated with shorter PFS. It is important to note that very few patients carried the A/A genotype which limits the power of the analysis. In addition, patients in the training cohort received cetuximab monotherapy or cetuximab combined with irinotecan, while patients in the Japanese cohort received cetuximab combined with a fluoropyrimidine (5-FU or S-1) and oxaliplatin. Each cytotoxic agent has its own immunomodulatory effects or may impact those seen with cetuximab, and this may have influenced our results. Nonetheless, our findings warrant further evaluation. If confirmed in larger, prospective studies, *CD24* rs52812045 genotype may be used to select mCRC patients most likely to benefit from cetuximab-based regimens in clinical practice. 

Another critical modulator of intestinal immunity and stem cell signaling is IDO1, an enzyme which catalyzes the rate-limiting step of tryptophan catabolism within the kynurenine pathway [39]. Released by tumor and myeloid derived suppressor cells, IDO1 depletes T-cells of tryptophan, thereby attenuating their activity [40]. Plasmacytoid dendritic cells express IDO1 to convert naïve T-cells into T_reg_-cells and increase PD-L1/PD-L2 expression, leading to T-cell suppression [41,42]. Independent of its immunoregulatory effects, IDO1 has been shown to activate canonical Wnt-β-catenin signaling and promote tumor progression by decreasing the tryptophan/kynurenine ratio [43]. 

Clinically, IDO1 mRNA expression has been correlated with colitis severity [44], peri-tumoral immune tolerance, and worse survival in CRC patients [45,46]. *IDO1* genetic variants have been associated with IBD severity [47], and we previously demonstrated the prognostic value of *IDO1* rs3739319 in patients with liver-limited mCRC [48]. Consistent with prior findings, the *IDO1* rs3739319 A allele was a negative prognosticator in our training cohort, though an opposite relationship was observed in the FIRE-3 validation cohort. Notably, the *IDO1* rs9657182 T allele conferred a positive prognostic effect in patients receiving irinotecan-cetuximab in the training cohort, but a negative effect in patients receiving oxaliplatin-cetuximab regimens in the Japanese validation cohort, and bevacizumab-treated patients of the FIRE-3 control cohort. Similarly, *PD-L1* rs2297137 predicted outcomes in both cetuximab and bevacizumab-treated patients. This may reflect the immunomodulatory activity of bevacizumab [49] and suggests distinct roles for IDO1 and PD-L1 in mediating these effects, which may vary by chemotherapy regimen. 

Our study has its limitations, the first being its retrospective nature. Prospective studies are needed to further validate our findings in patients with mCRC receiving cetuximab-based therapy. Clinical trials combining cetuximab with immune therapy and chemotherapy in patients with mCRC are ongoing and incorporating the *CD24* or *IDO1* genotype as a stratification factor may guide the use of these SNPs as predictive markers. Notably, there were certain genotypes that were carried by a small number of patients, and this limited our ability to test combinations of alleles and limits the strength of our conclusions. The heterogeneity of concomitant chemotherapy received between the cohorts may have also impacted our results, and future studies controlling for this variable will be important to translate the utility of these SNPs in the clinic. In addition, the *RAS* mutation status was unknown for a proportion of patients. As the role of *RAS* signaling on cetuximab-mediated ADCC is unclear [16,50,51,52], deciphering differential effects of these SNPs based on the *RAS* status could guide future study design. 

Studies examining the effect of these SNPs on gene function as well as associations between genotype/haplotype and immune cell subsets, serum cytokine levels, PDL-1 status, tumor mutational burden, and pathologic features (e.g., poor differentiation, tumor infiltrating lymphocytes, mucinous histology) may provide mechanistic explanations for the observed findings and better characterize the link between genotype and clinical outcomes. The presence of microsatellite instability (MSI) and *BRAF* V600E mutations also affect the tumor immune milieu and may have influenced the relationship between germline variants and outcomes; this information was not available in the present study. Therefore, further investigations should evaluate the effect of MSI and *BRAF* mutation status (V600E and perhaps non-V600E) on the predictive and prognostic utility of *CD24* or *IDO1* genotype, especially as it pertains to the efficacy of immune therapy combinations [53]. For instance, avelumab is currently being tested in combination with FOLFOXIRI plus cetuximab in the mCRC frontline setting in the phase 2 AVETRIC trial (NCT04513951). Others are examining the efficacy of first-line FOLFIRI plus cetuximab in mCRC patients based on FCγ-receptor genotype in the phase 2 CIFRA trial (NCT03874026). Specific germline polymorphisms or composite haplotypes, in conjunction with tumor features (e.g., sidedness) and molecular profile (e.g., *RAS*, *BRAF*, *HER2*, MSI status, CMS subtype) may serve to inform the selection of mCRC patients to treat with cetuximab and other targeted or immune therapies.

## 4. Materials and Methods

### 4.1. Study Design and Patient Population

A total of 924 mCRC patients with sufficient tissue for analysis were included in this study. The training cohort consisted of 105 patients enrolled in two phase II clinical trials. Thirty-two patients with EGFR-expressing mCRC refractory to irinotecan, oxaliplatin, and fluoropyrimidines were recruited October 2002-March 2003, at the University of Southern California/Norris Comprehensive Cancer Center (USC/NCCC) to the phase 2, open-label, multicenter ImClone-0144 (IMCL-0144) study [8], and treated with single-agent cetuximab. The remaining 73 patients were recruited to a phase 2 trial (protocol ASL608LIOM01) conducted by the Gruppo Oncologico Nord Ovest (EudraCT 2008-003160-19) and received irinotecan-cetuximab.

Among the twelve SNPs tested in the training cohort, five (*CD24* rs52812045, *IDO1* rs9657182 and rs3739319, *PDL1* rs2297137, *CTLA4* rs231777) showed significant associations with response rate (RR), progression-free survival (PFS), and/or overall survival (OS). These five SNPs were tested in two independent validation and two independent control cohorts. The first validation cohort consisted of 225 patients with *KRAS* exon 2 wildtype mCRC treated with FOLFIRI-cetuximab in FIRE-3 (NCT00433927). FIRE-3 was a phase 3 trial randomizing patients with mCRC to first-line FOLFIRI-cetuximab versus FOLFIRI-bevacizumab. The second validation cohort consisted of 74 patients enrolled in two phase 2 trials of first-line therapy with cetuximab-oxaliplatin-containing chemotherapy, either mFOLFOX6 (JACCRO-CC-05; *n* = 57, UMIN000004197) or SOX (JACCRO-CC-06; *n* = 67, UMIN000007022). Two control cohorts consisted of patients treated with FOLFIRI-bevacizumab in FIRE-3 (*n* = 292) and TRIBE (*n* = 228) (NCT00719797) [2,9]. TRIBE was a phase 3 trial randomizing patients with mCRC to first-line FOLFOXIRI-bevacizumab versus FOLFIRI-bevacizumab. The *KRAS* status was analyzed in 407 (80.1%) patients from TRIBE, of which 38.7% were wildtype in the FOLFIRI-bevacizumab arm [9].

Eligible patients had stage 4 histologically confirmed colorectal adenocarcinoma, with measurable disease per response evaluation criteria in solid tumors (RECIST) 1.0 criteria, and life expectancy of ≥12 weeks. Standard inclusion and exclusion criteria were applied. Treatment was administered until disease progression, intolerable toxicities, or patient withdrawal. The current study was conducted at the USC/NCCC and approved by the USC Institutional Review Board (HS-997018). All patients signed informed consent for molecular correlates analysis. This study adheres to the reporting recommendations for tumor MARKer prognostic studies (REMARK).

### 4.2. Candidate Single-Nucleotide Polymorphisms

Polymorphisms were selected based on a minimum allele frequency of 10% in Caucasians, functional or predicted functional relevance of the respective gene or protein, TagSNP, and location. Functional significance was predicted based on information derived from the National Institute of Environmental Health Sciences SNP Function Prediction [54], and the Queen’s University F-SNP [55]. Selected polymorphisms are presented in Table S1.

### 4.3. Genotyping

Genotyping was performed on DNA extracted from whole blood or tissue samples using the QIAamp Kit (Qiagen, Germantown, MD, USA), followed by PCR-based direct sequencing as previously published [56,57]. All primers (Table S1) were validated on known DNA sequences. Sequencing results were analyzed using the ABI Sequencing Scanner v1.0 (Applied Biosystems, Foster, CA, USA) by one investigator (N.B.V.) blinded to the patients’ identifying data and outcomes.

### 4.4. Statistical Analyses

The primary endpoint of this retrospective correlative analysis was OS, defined as the period from the randomization start date to death from any cause, or the date of last follow-up if patients were still alive. The secondary endpoints included RR per RECIST 1.0 and PFS. PFS was defined as the period from the randomization start date to first observation of tumor progression or death, whichever came first. PFS was censored at the date of last follow-up if patients remained alive and progression-free.

Patients were compared between cohorts using the Kruskal-Wallis test for continuous variables, chi-square test for categorical variables, and log-rank test for PFS and OS. Associations between SNPs and clinical outcomes were tested in univariable and multivariable analyses. The log-rank test and Kaplan-Meier curves were performed to investigate associations between SNPs and OS or PFS in univariable analysis. Multivariable Cox proportional hazard regression models were used to re-examine associations between SNPs and OS or PFS when adjusting for patient baseline characteristics. Fisher’s exact test was used to test associations between SNPs and RR. SNPs without significant associations with clinical outcomes are outlined in Tables S2–S6.

SAS 9.3 (SAS Institute, Cary, NC, USA) was used to perform all analyses. Case-wise deletion was applied when patients with missing SNPs were excluded in the analyses. All tests were two-sided at a significance level of 0.05.

## 5. Conclusions

Cetuximab is an established therapy for mCRC, which affects both EGFR signaling and ADCC. As the role of immune checkpoint inhibition in mCRC is being actively defined, identifying biomarkers of the immunomodulatory effects of cetuximab may refine its clinical use and inform the development of chemo-immunotherapy combinations. We examined common genetic variants within immune regulatory pathways and identified *CD24* and *IDO1* polymorphisms, which independently predicted outcomes. Further studies are needed to evaluate the functional relevance and clinical utility of these genetic variants in patients with mCRC receiving cetuximab-based therapy.

## Figures and Tables

**Table 1:** Baseline patient and tumor characteristics.

Characteristic	Training Cohort (*N* = 105)	FIRE-3 Validation Cohort 1 (*N* = 225)	Japanese Validation Cohort 2 (*N* = 74)	FIRE-3 Control Cohort 1 (*N* = 292)	TRIBE Control Cohort 2 (*N* = 228)	*p*-Value *
Age, years						
Median (range)	63 (35–83)	64 (38–79)	63 (40-79)	65 (31-76)	60 (29–75)	<0.001
Sex						
Male	62 (59%)	159 (71%)	42 (57%)	194 (66%)	138 (61%)	0.059
Female	43 (41%)	66 (29%)	32 (43%)	98 (34%)	90 (39%)	
Overall Response Rate (ORR)	20.0%	61.3%	71.6%	57.2%	56.6%	<0.001
Disease Control Rate (DCR)	61.9%	80.0%	91.9%	83.2%	89.0%	<0.001
Median PFS, months ** (95% CI)	3.7 (3.2–4.9)	9.8 (8·7–10.8)	10.0 (8.9–13.7)	10.1 (9.6–11.2)	9·7 (9.2–10.8)	<0.001
Median OS, months ** (95% CI)	10.6 (7.8–13.2)	28.6 (23.7–36.3)	33.9 (26.5+)	23.7 (21.4–26.4)	26.1 (22.7-30.9)	<0.001

* *p*-value was based on the Kruskal-Wallis test for age, chi-square test for categorical variables, and log-rank test for PFS and OS. ** Median follow-up was 18.2 months (range: 1.2–42.4 months), 35.3 months (range: 0.0–70.7 months), 31.0 months (range: 5.4–42.9 months), 40.7 months (range: 0.0–69.7 months), and 33.5 months (range: 2.8–53.2 months) in the training, validation 1, validation 2, FIRE-3 control, and TRIBE control cohorts, respectively.

**Table 2:** Immune regulatory single nucleotide polymorphisms (SNPs) and outcomes in patients with advanced colorectal cancer (CRC) treated with cetuximab-based therapy (training cohort, Italian + USC).

SNP		Tumor Response		Progression-Free Survival			Overall Survival		
	*N*	PR	SD + PD	Median, ms (95% CI)	Univariable HR (95% CI) ^†^	Multivariable HR (95% CI) ^‡^	Median, ms (95% CI)	Univariable HR (95% CI) ^†^	Multivariable HR (95% CI) ^‡^
*IDO1* rs9657182									
C/C	25	3 (12%)	22 (88%)	3.3 (2.2, 4.4)	1 (reference)	1 (reference)	8.5 (5.1, 10.8)	1 (reference)	1 (reference)
C/T	55	11 (20%)	43 (80%)	4.6 (3.4, 5.7)	0.77 (0.47, 1.26)	0.71 (0.42, 1.19)	15.0 (7.8, 15.9)	0.49 (0.27, 0.89)	0.39 (0.20, 0.74)
T/T	24	7 (30%)	16 (70%)	4.1 (2.4, 7.4)	0.56 (0.30, 1.04)	0.47 (0.25, 0.89)	12.0 (5.7, 20.4)	0.54 (0.27, 1.07)	0.38 (0.18, 0.79)
*p*-value *			0.33		0.13	0.068		0.021	0.008
*IDO1* rs3739319									
G/G	27	8 (31%)	18 (69%)	5.2 (4.0, 8.0)	1 (reference)	1 (reference)	17.9 (8.5, 26.4)	1 (reference)	1 (reference)
A/G	51	9 (18%)	41 (82%)	3.6 (2.5, 5.2)	1.35 (0.82, 2.23)	1.55 (0.92, 2.60)	8.7 (6.3, 12.0)	2.12 (1.15, 3.93)	2.32 (1.20, 4.50)
A/A	27	4 (15%)	23 (85%)	2.8 (2.4, 4.7)	1.72 (0.97, 3.08)	1.55 (0.83, 2.88)	10.8 (4.4, 15.0)	2.13 (1.04, 4.36)	2.06 (0.95, 4.47)
*p*-value *			0.32		0.15	0.22		0.028	0.043
*CD24* rs52812045									
G/G	54	8 (15%)	45 (85%)	3.6 (2.5, 4.6)	1 (reference)	1 (reference)	7.8 (5.7, 10.5)	1 (reference)	1 (reference)
G/A	36	10 (29%)	25 (71%)	5.0 (3.3, 6.6)	0.74 (0.47, 1.16)	0.62 (0.39, 1.00)	13.1 (8.7, 17.9)	0.58 (0.34, 0.99)	0.50 (0.28, 0.88)
A/A	5	0 (%)	5 (100%)	1.3 (1.0, 3.3)	3.62 (1.35, 9.70)	3.18 (1.10, 9.20)	2.3 (1.8, 11.3)	3.27 (1.23, 8.68)	4.93 (1.65, 14.77)
*p*-value *			0.20		0.001	0.009		<0.001	0.001

* *p*-value was based on Fisher’s exact test for response, log-rank test for PFS and OS in the univariate analysis (^†^), and Wald test for PFS and OS in the multivariable Cox regression model (^‡^) that was adjusted for sex, age, rash, and racial background.

**Table 3:** Immune regulatory SNPs and outcomes in patients receiving first-line FOLFIRI and cetuximab in FIRE-3 (validation cohort 1).

SNP		Tumor Response		Progression-Free Survival			Overall Survival		
	*N*	PR	PD + SD	Median, ms (95% CI)	Univariable HR (95% CI)	Multivariable HR (95% CI)	Median, ms (95% CI)	Univariable HR (95% CI)	Multivariable HR (95% CI)
*IDO1* rs9657182									
C/C	63	39 (70%)	17 (30%)	9.6 (8.2, 13.0)	1 (reference)	1 (reference)	28.7 (16.8, 38.3)	1 (reference)	1 (reference)
C/T	86	48 (70%)	21 (30%)	9.7 (6.9, 11.3)	1.18 (0.83, 1.69)	1.09 (0.74, 1.61)	25.2 (22.6, 33.6)	1.15 (0.75, 1.76)	0.90 (0.56, 1.43)
T/T	72	49 (73%)	18 (27%)	10.0 (7.9, 11.5)	1.06 (0.74, 1.54)	1.13 (0.76, 1.68)	36.6 (21.3, 52.0)	0.84 (0.53, 1.32)	0.73 (0.45, 1.17)
*p*-value *			0.88		0.64	0.83		0.34	0.41
C/T or T/T	158	97 (71%)	39 (29%)	10.0 (8.2, 10.9)	1.12 (0.81, 1.55)	1.11 (0.78, 1.57)	28.0 (23.7, 37.5)	0.99 (0.67, 1.45)	0.81 (0.54, 1.22)
*p*-value *			0.86		0.48	0.56		0.96	0.32
*IDO1* rs3739319									
G/G	94	54 (69%)	24 (31%)	9.2 (7.9, 10.6)	1 (reference)	1 (reference)	23.9 (20.6, 36.6)	1 (reference)	1 (reference)
G/A	78	50 (69%)	22 (31%)	10.3 (7.8, 12.6)	0.81 (0.59, 1.12)	0.68 (0.48, 0.97)	30.6 (22.6, 49.8)	0.83 (0.56, 1.23)	0.75 (0.49, 1.16)
A/A	46	29 (74%)	10 (26%)	12.1 (8.7, 14.1)	0.68 (0.46, 1.00)	0.67 (0.45, 0.99)	38.7 (27.9, 68.7)	0.57 (0.35, 0.94)	0.61 (0.36, 1.04)
*p*-value *			0.88		0.12	0.040		0.079	0.14
G/A or A/A	124	79 (71%)	32 (29%)	10.5 (8.8, 12.6)	0.76 (0.57, 1.02)	0.67 (0.50, 0.91)	33.1 (27.6, 41.2)	0.72 (0.50, 1.03)	0.70 (0.48, 1.02)
*p*-value *			0.87		0.059	0.011		0.068	0.063
*CD24* rs52812045									
G/G	137	84 (72%)	33 (28%)	9.3 (7.9, 10.6)	1 (reference)	1 (reference)	25.2 (20.6, 33.4)	1 (reference)	1 (reference)
G/A	66	43 (68%)	20 (32%)	10.4 (8.0, 12.3)	0.92 (0.67, 1.27)	1.01 (0.72, 1.44)	33.8 (24.5, 40.0)	0.79 (0.53, 1.18)	0.61 (0.38, 0.97)
A/A	11	4 (57%)	3 (43%)	14.1 (5.7, 19.9)	0.61 (0.32, 1.17)	0.54 (0.26, 1.11)	41.2 (19.9, 56.2)	0.72 (0.33, 1.56)	1.15 (0.50, 2.65)
*p*-value *			0.56		0.31	0.24		0.40	0.093
G/A or A/A	77	47 (67%)	23 (33%)	11.3 (9.0, 12.8)	0.86 (0.64, 1.16)	0.90 (0.65, 1.25)	37.5 (26.5, 41.2)	0.77 (0.53, 1.13)	0.68 (0.44, 1.04)
*p*-value *			0.51		0.32	0.55		0.18	0.074

* *p*-value was based on Fisher’s exact test for tumor response, log-rank test for PFS and OS in the univariable analysis, and Wald test in the multivariable Cox proportional hazards regression model adjusting for sex, primary tumor site, liver metastases, number of metastatic sites, LDH, Kohne score, RAS and BRAF mutation status.

**Table 4:** Immune regulatory SNPs and outcomes in Japanese patients receiving first-line cetuximab plus oxaliplatin-based therapy (validation cohort 2).

SNP		Tumor Response		Progression-Free Survival			Overall Survival		
	*N*	PR	SD + PD	Median, ms (95% CI)	Univariable HR (95% CI) ^†^	Multivariable HR (95% CI) ^‡^	Median, ms (95% CI)	Univariable HR (95% CI) ^†^	Multivariable HR (95% CI) ^‡^
*IDO1* rs9657182									
C/C	26	19 (79%)	5 (21%)	11.6 (9.2, 15.2)	1 (reference)	1 (reference)	41.1+ (17.9, 41.1+)	1 (reference)	1 (reference)
C/T	30	19 (68%)	9 (32%)	9.5 (5.6, 13.8)	1.14 (0.63, 2.06)	1.11 (0.61, 2.04)	26.5 (18.4, 36.2)	2.18 (0.98, 4.85)	1.98 (0.86, 4.56)
T/T	16	13 (81%)	3 (19%)	9.1 (5.1, 16.7)	1.35 (0.66, 2.76)	1.21 (0.57, 2.55)	42.9+ (27.1, 42.9+)	0.92 (0.33, 2.58)	0.87 (0.31, 2.47)
*p*-value *			0.63		0.68	0.88		0.049	0.12
C/T or T/T	46	32 (73%)	12 (27%)	9.5 (8.0, 13.6)	1.21 (0.70, 2.07)	1.14 (0.65, 2.00)	27.5 (23.2, 42.8)	1.64 (0.76, 3.52)	1.47 (0.66, 3.25)
*p*-value *			0.77		0.48	0.65		0.19	0.34
*IDO1* rs3739319									
G/G	22	17 (81%)	4 (19%)	10.0 (8.9, 15.2)	1 (reference)	1 (reference)	27.1 (16.0, 42.8)	1 (reference)	1 (reference)
G/A	34	21 (66%)	11 (34%)	9.1 (6.1, 11.8)	0.96 (0.52, 1.75)	0.98 (0.53, 1.81)	26.7 (18.4, 42.9)	0.98 (0.47, 2.05)	0.94 (0.45, 1.98)
A/A	16	13 (87%)	2 (13%)	13.6 (5.6, 15.2)	0.79 (0.37, 1.69)	0.75 (0.35, 1.64)	41.4+ (23.5, 41.4+)	0.53 (0.20, 1.41)	0.51 (0.19, 1.39)
*p*-value *			0.27		0.80	0.73		0.35	0.38
G/A or A/A	50	34 (72%)	13 (28%)	9.7 (8.0, 13.8)	0.90 (0.51, 1.59)	0.91 (0.51, 1.62)	36.2 (21.5, 42.9)	0.80 (0.40, 1.61)	0.78 (0.39, 1.58)
*p*-value *			0.55		0.71	0.74		0.52	0.49
*CD24* rs52812045									
G/G	34	23 (74%)	8 (26%)	11.8 (9.4, 18.9)	1 (reference)	1 (reference)	33.9 (19.0, 39.6)	1 (reference)	1 (reference)
G/A ^a^	28	21 (78%)	6 (22%)	9.2 (6.7, 13.8)	1.90 (1.05, 3.42)	2.12 (1.14, 3.95)	36.2 (23.5, 42.9)	0.88 (0.44, 1.76)	0.85 (0.41, 1.76)
A/A ^a^	6	5 (83%)	1 (17%)						
*p*-value *			1.00		0.011	0.018		0.71	0.66

* *p*-value was based on Fisher’s exact test for response, log-rank test for PFS and OS in the univariable analysis (^†^), and Wald test for PFS and OS in the multivariable Cox regression model (^‡^) adjusting for ECOG performance status (0 vs. 1), and chemo backbone (FOLFOX vs. SOX). ^a^ Grouped together to estimate hazard ratios.

**Table 5:** Immune regulatory SNPs and outcomes in patients treated with first-line FOLFIRI and bevacizumab in FIRE-3 (control cohort 1).

SNP		Tumor Response		Progression-Free Survival			Overall Survival		
	*N*	PR	SD + PD	Median, ms (95% CI)	Univariable HR (95% CI) ^†^	Multivariable HR (95% CI) ^‡^	Median, ms (95% CI)	Univariable HR (95% CI) ^†^	Multivariable HR (95% CI) ^‡^
*IDO1* rs9657182									
C/C	76	47 (66%)	24 (34%)	13.2 (10.4, 13.9)	1 (reference)	1 (reference)	26.4 (21.3, 32.8)	1 (reference)	1 (reference)
C/T	112	65 (62%)	40 (38%)	9.2 (8.6, 10.5)	1.61 (1.17, 2.22)	1.58 (1.12, 2.21)	22.1 (18.4, 26.3)	1.28 (0.89, 1.83)	1.28 (0.88, 1.87)
T/T	74	39 (57%)	29 (43%)	10.1 (8.8, 11.8)	1.26 (0.89, 1.79)	1.29 (0.90, 1.85)	24.8 (20.6, 29.1)	0.98 (0.66, 1.46)	1.21 (0.81, 1.82)
*p*-value *			0.58		0.010	0.031		0.22	0.42
C/T or T/T	186	104 (60%)	69 (40%)	9.8 (8.9, 10.5)	1.45 (1.08, 1.95)	1.44 (1.06, 1.96)	23.1 (20.1, 26.7)	1.14 (0.82, 1.59)	1.25 (0.89, 1.76)
*p*-value *			0.39		0.010	0.019		0.43	0.20
*IDO1* rs3739319									
G/G	94	51 (61%)	33 (39%)	9.9 (8.6, 11.5)	1 (reference)	1 (reference)	21.9 (17.5, 25.6)	1 (reference)	1 (reference)
G/A	129	75 (62%)	46 (38%)	9.7 (8.8, 11.3)	0.87 (0.65, 1.16)	0.85 (0.63, 1.15)	25.0 (21.5, 28.4)	0.89 (0.64, 1.22)	0.87 (0.62, 1.22)
A/A	47	30 (68%)	14 (32%)	11.9 (9.8, 14.1)	0.75 (0.52, 1.10)	0.80 (0.54, 1.18)	27.6 (18.5, 35.0)	0.82 (0.53, 1.25)	0.89 (0.57, 1.38)
*p*-value *			0.72		0.30	0.43		0.59	0.71
G/A or A/A	176	105 (64%)	60 (36%)	10.3 (9.2, 11.8)	0.83 (0.63, 1.09)	0.83 (0.63, 1.10)	25.1 (22.3, 28.6)	0.87 (0.64, 1.17)	0.88 (0.64, 1.20)
*p*-value *			0.68		0.18	0.21		0.34	0.41
*CD24* rs52812045									
G/G	130	68 (56%)	53 (44%)	9.6 (8.8, 11.7)	1 (reference)	1 (reference)	23.7 (19.4, 27.4)	1 (reference)	1 (reference)
G/A ^a^	129	79 (66%)	40 (34%)	10.5 (9.8, 12.4)	0.86 (0.66, 1.12)	0.77 (0.59, 1.02)	26.1 (21.5, 28.8)	1.01 (0.76, 1.35)	1.00 (0.74, 1.36)
A/A ^a^	7	4 (57%)	3 (43%)						
*p*-value *			0.25		0.25	0.068		0.93	0.98

* *p*-value was based on Fisher’s exact test for tumor response, log-rank test for PFS and OS in the univariable analysis (^†^), and Wald test in the multivariable Cox regression model (^‡^) adjusting for sex, ECOG performance status, primary tumor resection, number of metastatic sites, adjuvant chemotherapy, LDH, RAS and BRAF mutation status. ^a^ Grouped together to estimate hazard ratios.

**Table 6:** Immune regulatory SNPs and outcomes in patients receiving first-line FOLFIRI and bevacizumab in TRIBE (control cohort 2).

SNP		Tumor Response		Progression-Free Survival			Overall Survival		
	*N*	PR	SD + PD	Median, ms (95% CI)	Univariable HR (95% CI) ^†^	Multivariable HR (95% CI) ^‡^	Median, ms (95% CI)	Univariable HR (95% CI) ^†^	Multivariable HR (95% CI) ^‡^
*IDO1* rs9657182									
C/C	44	20 (49%)	21 (51%)	8.8 (7.8, 10.5)	1 (reference)	1 (reference)	23.6 (14.7, 33.9)	1 (reference)	1 (reference)
C/T	112	66 (61%)	42 (39%)	9.4 (8.6, 10.5)	0.82 (0.55, 1.22)	0.87 (0.57, 1.32)	25.6 (21.1, 28.6)	0.94 (0.64, 1.40)	0.99 (0.65, 1.49)
T/T	65	38(58%)	27(42%)	11.3 (9.6, 12.7)	0.67 (0.44, 1.04)	0.70 (0.45, 1.11)	30.9 (22.7, 39.7)	0.74 (0.48, 1.14)	0.74 (0.47, 1.16)
*p*-value *			0.40		0.18	0.29		0.28	0.24
A/G or G/G	177	104 (60%)	69 (40%)	10.3 (9.3, 11.1)	0.76 (0.52, 1.10)	0.80 (0.54, 1.19)	26.2 (22.7, 30.8)	0.86 (0.59, 1.25)	0.88 (0.60, 1.30)
*p*-value *			0.22		0.14	0.27		0.42	0.52
*IDO1* rs3739319									
G/G	78	45 (60%)	30 (40%)	11.1 (8.3, 12.3)	1 (reference)	1 (reference)	23.9 (19.4, 28.7)	1 (reference)	1 (reference)
G/A	90	44 (50%)	44 (50%)	9.4 (8.7, 10.4)	1.19 (0.84, 1.69)	1.25 (0.87, 1.80)	26.5 (22.0, 33.0)	0.82 (0.59, 1.16)	0.84 (0.59, 1.20)
A/A	48	33 (70%)	14 (30%)	9.7 (8.6, 10.8)	1.01 (0.67, 1.55)	1.12 (0.71, 1.76)	29.2 (19.6, 39.2)	0.71 (0.47, 1.08)	0.80 (0.52, 1.23)
*p*-value *			0.070		0.55	0.48		0.25	0.50
G/A or A/A	138	77 (57%)	58 (43%)	9.5 (9.0, 10.4)	1.13 (0.82, 1.55)	1.20 (0.87, 1.67)	27.4 (23.9, 33.6)	0.78 (0.57, 1.07)	0.83 (0.60, 1.14)
*p*-value *			0.77		0.45	0.27		0.12	0.24
*CD24* rs52812045									
G/G	123	71 (59%)	49 (41%)	9.5 (8.8, 10.8)	1 (reference)	1 (reference)	28.7 (23.9, 35.1)	1 (reference)	1 (reference)
G/A	79	42 (55%)	34 (45%)	9.9 (9.3, 12.6)	0.98 (0.71, 1.35)	0.78 (0.55, 1.10)	24.0 (20.3, 27.3)	1.30 (0.94, 1.79)	1.38 (0.99, 1.94)
A/A	21	13 (62%)	8 (38%)	9.2 (6.7, 11.3)	1.22 (0.71, 2.11)	1.00 (0.56, 1.79)	22.3 (15.6, 48.7)	1.17 (0.70, 1.97)	1.34 (0.78, 2.30)
*p*-value *			0.82		0.73	0.35		0.27	0.14
G/A or A/A	100	55 (57%)	42 (43%)	9.7 (9.2, 11.6)	1.02 (0.75, 1.38)	0.82 (0.59, 1.13)	23.5 (20.3, 27.3)	1.27 (0.94, 1.71)	1.38 (1.00, 1.89)
*p*-value *			0.78		0.91	0.22		0.11	0.049

* *p*-value was based on Fisher’s exact test for tumor response, log-rank test for PFS and OS in the univariable analysis (**^†^**), and Wald test in the multivariable Cox proportional hazards regression model (**^‡^**) adjusting for sex, age, ECOG performance status, primary tumor site, number of metastatic sites, BRAF mutation status, resection of the primary tumors, and adjuvant therapy.

## Supplementary Materials

The following are available online at https://www.mdpi.com/2072-6694/12/10/2947/s1, Table S1: Candidate single-nucleotide polymorphisms (SNPs); Table S2: Immune regulatory SNPs and outcomes in patients with advanced CRC treated with cetuximab-based therapy (training cohort, Italian + USC); Table S3: Immune regulatory SNPs and outcomes in patients receiving first-line FOLFIRI and cetuximab in FIRE-3 (validation cohort 1); Table S4: Immune regulatory SNPs and outcomes in Japanese patients receiving first-line cetuximab plus oxaliplatin-based therapy (validation cohort 2); Table S5: Immune regulatory SNPs and outcomes in patients treated with first-line FOLFIRI and bevacizumab in FIRE-3 (control cohort 1); Table S6: Immune regulatory SNPs and outcomes in patients receiving first-line FOLFIRI and bevacizumab in TRIBE (control cohort 2).
