# Supplementary material for: Polymorphisms within Immune Regulatory Pathways Predict Cetuximab Efficacy and Survival in Metastatic Colorectal Cancer Patients
Source: Cancers (Basel). 2020 Oct 13;12(10):2947. doi: 10.3390/cancers12102947 (PMC7601940; doi:10.3390/cancers12102947)
Supplement: Supplementary file 1 [file cancers-12-02947-s001.pdf]

Polymorphisms within Immune Regulatory Pathways Predict Cetuximab Efficacy and Survival in Metastatic Colorectal Cancer Patients

Nico B. Volz, Diana L. Hanna, Sebastian Stintzing, Wu Zhang, Dongyun Yang, Shu Cao, Yan Ning, Satoshi Matsusaka, Yu Sunakawa, Martin D. Berger, Chiara Cremolini, Fotios Loupakis, Alfredo Falcone and Heinz-Josef Lenz

Table S1. Candidate single-nucleotide polymorphisms (SNPs).

Gene	Genotype	Function	Allele Frequency	Forward (F) and Reverse (R) Primers
CD24				
rs52812045	G/A	unknown	G: NA [#]	F: AAC TAA TGC CAC CAC CAA GG
			A: NA [#]	R: CCT GTT TTT CCT TGC CACA T
IDO1				
rs9657182	T/C	unknown	T: 55%	F: GGT CAT AAA AGG AGA AAA TGA CC
			C: 45%	R: TTG CAA AGA GCT TTC AGA AAA A
rs3739319	G/A	transcriptional regulation	G: 60%	F: GAC CTT GAC CTC AGT GAA TGC
			A: 49%	R: ATG TCC TGG AGG AAC TGA GC
rs10108662	C/A		C: 68%	F: CTT GGA CAA TGG GCC TAA AA
			A: 32%	R: GGA AGG CAA GAA GTT TAG CAA
PD1				
rs2227981	G/A	splicing regulation	G: 60%	F: AGG GTC TGC AGA ACA CTG GT
			A: 40%	R: TCC CTG AGC AGA CGG AGT AT
rs7421861	A/G	unknown	A: 66%	F: AAG TCT CTG CTG GGG CCT CT
			G: 34%	R: GGA AGG GGG TGA AGG CTC T
PDL1				
rs2297137	G/A	transcriptional regulation	G: 73%	F: AAG GGG TGA GAA TTG GAT CA
			A: 27%	R: ATT CTC CTG CCC CTC ACC
rs2297136	A/G		A: 54%	F: TCC AGC ATT GGA ACT TCT GA
			G: 46%	R: CCA GGT TCC ATT TTC AGT GC
rs10122089	T/C		T: 54%	F: GAA CAT GAG GCT GTG TGT CC
			C: 46%	R: TTG AAT TTG TGC AGG CAA TG
no rs number	G/C	increases CD274 expression	G: NA [#]	F: AAA TCA TCC ATT GCT CAT CCT
			C: NA [#]	R: CAT AAA TAC TGT CCC GTT CCA A
CTLA4				
rs231777	C/T	transcriptional regulation	C: 85%	F: TGA CAC CAG GTT TGT TAC ACG
			T:15%	R: TCT TCT CCA GTA AAA ATT AAA TCA TCA
rs231775	A/G	splicing regulation	A: 65%	F: ACT TCC TGA AGA CCT GAA CAC C
			G: 36%	R: CTC ACC TTT GCA GAA GAC AGG

[#] Frequency not reported in the literature or public database. In our study, CD274: G: 65% and C: 35%; CD24 rs52812045 C: 76% and T: 24%.

Table S2. Immune regulatory SNPs and outcomes in patients with advanced CRC treated with cetuximab-based therapy (Training Cohort, Italian + USC).

		Tumor Response		Progression-Free Survival			Overall Survival		
	N	PR	SD+PD	Median, ms (95% CI)	Univariable HR (95% CI) †	Multivariable HR (95% CI) ‡	Median, ms (95% CI)	Univariable HR (95% CI)†	Multivariable HR (95% CI)‡
IDO1 rs10108662									
C/C	52	9 (18%)	42 (82%)	3.7 (3.3, 5.2)	1 (reference)	1 (reference)	9.1 (7.0, 15.4)	1 (reference)	1 (reference)
C/A	39	9 (24%)	29 (76%)	3.7 (2.5, 5.7)	1.00 (0.65, 1.55)	0.90 (0.56, 1.43)	12.0 (7.2, 15.2)	0.88 (0.53, 1.46)	0.77 (0.43, 1.39)
A/A	14	3 (21%)	11 (79%)	3.0 (1.6, 8.7)	0.86 (0.45, 1.64)	0.99 (0.51, 1.93)	13.3 (2.2, 16.3)	1.03 (0.47, 2.22)	1.11 (0.50, 2.47)
P value *			0.75		0.88	0.89		0.86	0.60
PD1 rs2227981									
G/G	43	9 (21%)	33 (79%)	4.1 (2.5, 5.7)	1 (reference)	1 (reference)	12.1 (5.6, 13.5)	1 (reference)	1 (reference)
G/A	42	8 (20%)	33 (80%)	3.7 (3.0, 4.7)	1.16 (0.74, 1.82)	1.17 (0.74, 1.85)	9.0 (7.4, 11.3)	1.03 (0.62, 1.72)	1.16 (0.68, 1.98)
A/A	20	4 (20%)	16 (80%)	3.7 (2.2, 8.0)	0.75 (0.42, 1.32)	0.68 (0.38, 1.24)	15.2 (4.5, 26.9)	0.76 (0.39, 1.50)	0.83 (0.41, 1.68)
P value *			1.00		0.27	0.23		0.65	0.63
PD1 rs7421861									
A/A	48	12 (26%)	35 (74%)	3.7 (2.5, 6.5)	1 (reference)	1 (reference)	12.8 (7.2, 15.7)	1 (reference)	1 (reference)
A/G	41	4 (10%)	37 (90%)	3.7 (2.5, 4.7)	1.36 (0.87, 2.12)	1.46 (0.92, 2.31)	7.7 (5.1, 9.1)	1.51 (0.91, 2.51)	1.56 (0.91, 2.67)
G/G	14	4 (31%)	9 (69%)	5.2 (3.3, 8.7)	0.91 (0.47, 1.79)	1.10 (0.55, 2.22)	13.5 (7.8, 21.4)	0.97 (0.45, 2.12)	1.02 (0.45, 2.32)
P value *			0.088		0.26	0.26		0.20	0.23
PDL1 rs2297137									
G/G	60	9 (16%)	49 (84%)	4.1 (3.3, 5.2)	1 (reference)	1 (reference)	9.1 (7.4, 15.2)	1 (reference)	1 (reference)
G/A	36	7 (19%)	29 (81%)	3.4 (2.3, 5.0)	1.27 (0.82, 1.95)	1.30 (0.82, 2.05)	9.0 (4.4, 13.5)	1.45 (0.88, 2.37)	1.43 (0.82, 2.49)
A/A	9	5 (56%)	4 (44%)	7.4 (3.7, 11.3)	0.65 (0.29, 1.43)	0.49 (0.22, 1.11)	15.0 (2.2, 26.9)	0.63 (0.22, 1.76)	0.49 (0.17, 1.42)
P value *			**0.029**		0.22	0.079		0.15	0.14
PDL1 rs2297136									
A/A	43	9 (21%)	34 (79%)	2.8 (2.4, 4.6)	1 (reference)	1 (reference)	9.0 (4.8, 15.5)	1 (reference)	1 (reference)
A/G	46	11 (24%)	34 (76%)	4.7 (3.4, 7.0)	0.75 (0.48, 1.16)	0.70 (0.43, 1.16)	12.1 (7.4, 15.7)	0.95 (0.57, 1.61)	0.87 (0.49, 1.55)
G/G	15	1 (7%)	13 (93%)	4.4 (2.5, 6.1)	0.85 (0.46, 1.58)	0.75 (0.38, 1.45)	9.1 (3.4, 12.0)	1.20 (0.59, 2.42)	0.97 (0.45, 2.08)
P value *			0.42		0.41	0.37		0.80	0.89
PDL1 rs10122089									
T/T	38	7 (18%)	31 (82%)	2.6 (2.4, 4.6)	1 (reference)	1 (reference)	9.0 (4.8, 15.0)	1 (reference)	1 (reference)
T/C	50	12 (24%)	37 (76%)	4.7 (3.4, 7.0)	0.72 (0.46, 1.14)	0.92 (0.56, 1.53)	13.1 (7.8, 15.7)	0.94 (0.56, 1.59)	1.20 (0.66, 2.16)
C/C	16	2 (13%)	13 (87%)	4.1 (2.3, 5.3)	0.92 (0.50, 1.70)	1.18 (0.63, 2.23)	7.7 (3.4, 10.5)	1.32 (0.67, 2.61)	1.78 (0.88, 3.60)
P value *			0.67		0.32	0.72		0.59	0.28
PDL1 G_C									
G/G	40	6 (16%)	32 (84%)	4.7 (3.7, 6.8)	1 (reference)	1 (reference)	12.0 (8.4, 20.4)	1 (reference)	1 (reference)

G/C	55	12 (22%)	43 (78%)	3.3 (2.5, 4.6)	1.54 (0.99, 2.39)	1.58 (1.00, 2.49)	8.5 (5.1, 12.1)	1.64 (0.99, 2.72)	1.73 (1.01, 2.97)
C/C	8	3 (38%)	5 (63%)	4.6 (1.1, 11.3)	0.84 (0.36, 1.98)	0.72 (0.29, 1.80)	15.5 (2.2, 26.9)	0.89 (0.31, 2.55)	0.77 (0.26, 2.29)
P value *			0.37		0.066	0.072		0.094	0.093
CTLA4 rs231777									
C/C	65	13 (20%)	52 (80%)	4.1 (3.3, 5.3)	1 (reference)	1 (reference)	9.0 (7.7, 15.0)	1 (reference)	1 (reference)
C/T	34	6 (19%)	26 (81%)	2.6 (2.3, 4.4)	1.40 (0.90, 2.18)	1.76 (1.10, 2.81)	8.5 (4.5, 13.1)	1.13 (0.68, 1.87)	1.26 (0.75, 2.13)
P value *			1.00		0.12	0.019		0.62	0.38
CTLA4 rs231775									
A/A	57	12 (21%)	44 (79%)	4.1 (3.3, 5.6)	1 (reference)	1 (reference)	10.5 (7.7, 15.2)	1 (reference)	1 (reference)
A/G	39	8 (21%)	30 (79%)	4.0 (2.6, 5.3)	1.20 (0.78, 1.85)	0.99 (0.62, 1.58)	12.1 (7.3, 15.4)	1.17 (0.71, 1.92)	1.01 (0.59, 1.74)
G/G	7	1 (14%)	6 (86%)	2.4 (1.0, 6.2)	2.24 (0.87, 5.78)	0.75 (0.16, 3.57)	5.7 (2.2, 8.5)	2.34 (0.80, 6.87)	0.54 (0.10, 3.05)
P value *			1.00		0.17	0.94		0.24	0.77

* *P* value was based on Fisher's exact test for response, log-rank test for PFS and OS in the univariate analysis (†) and Wald test for PFS and OS in the multivariable Cox regression model (‡) that was adjusted for sex, age, rash, and racial background.

Table S3. Immune regulatory SNPs and outcomes in patients receiving first-line FOLFIRI and cetuximab in FIRE-3 (Validation Cohort 1).

	Tumor Response			Progression-Free Survival			Overall Survival		
	N	**PR**	**SD + PD**	**Median, ms (95% CI)**	**Univariable HR (95% CI)**	**Multivariable HR (95% CI)**	**Median, ms (95% CI)**	**Univariable HR (95% CI)**	**Multivariable HR (95% CI)**
PDL1 rs2297137									
G/G	120	69 (67%)	34 (33%)	10.4 (8.7, 12.8)	1 (reference)	1 (reference)	29.8 (23.7, 40.0)	1 (reference)	1 (reference)
G/A	72	48 (77%)	14 (23%)	9.5 (7.8, 10.9)	1.26 (0.92, 1.74)	1.15 (0.82, 1.61)	33.1 (20.6, 38.7)	0.96 (0.65, 1.42)	1.00 (0.67, 1.51)
A/A	22	13 (68%)	6 (32%)	10.0 (6.9, 12.2)	1.22 (0.74, 2.00)	1.44 (0.85, 2.45)	25.2 (14.8, 59.0)	1.23 (0.66, 2.29)	1.34 (0.68, 2.63)
P value *			0.34		0.31	0.35		0.75	0.68
G/A or A/A	94	61 (75%)	20 (25%)	9.5 (7.8, 10.6)	1.25 (0.93, 1.68)	1.21 (0.88, 1.65)	27.1 (21.7, 38.3)	1.01 (0.70, 1.46)	1.06 (0.72, 1.56)
P value *			0.26		0.13	0.23		0.95	0.77
CTLA4 rs231777									
C/C	171	100 (68%)	46 (32%)	9.7 (8.3, 10.9)	1 (reference)	1 (reference)	33.4 (24.5, 40.0)	1 (reference)	1 (reference)
C/T [a]	45	30 (75%)	10 (25%)	10.4 (7.9, 13.5)	0.89 (0.62, 1.27)	1.00 (0.67, 1.50)	23.9 (17.5, 29.8)	1.32 (0.86, 2.03)	1.39 (0.88, 2.19)
T/T [a]	3	3 (100%)	0 (0%)						
P value *			0.55		0.52	0.98		0.20	0.16

* *P* value was based on Fisher's exact test for tumor response, log-rank test for PFS and OS in the univariable analysis, and Wald test in the multivariable Cox proportional hazards regression model adjusting for sex, primary tumor site, liver metastases, number of metastatic sites, LDH, Kohne score, RAS and BRAF mutation status. [a] Grouped together to estimate hazard ratios.

Table S4. Immune regulatory SNPs and outcomes in Japanese patients receiving first-line cetuximab plus oxaliplatin-based therapy (Validation Cohort 2).

	Tumor Response			Progression-Free Survival			Overall Survival		
	N	PR	SD + PD	Median, ms (95% CI)	Univariable HR (95% CI)†	Multivariable HR (95% CI) ‡	Median, ms (95% CI)	Univariable HR (95% CI) †	Multivariable HR (95% CI)‡
PDL1 rs2297137									
G/G	19	11 (69%)	5 (31%)	11.8 (9.4, 16.8)	1 (reference)	1 (reference)	40.3+ (14.9, 40.3+)	1 (reference)	1 (reference)
G/A	36	27 (75%)	9 (25%)	9.2 (6.1, 13.6)	1.29 (0.69, 2.42)	1.20 (0.63, 2.29)	30.5 (18.4, 42.9)	1.12 (0.48, 2.60)	0.95 (0.39, 2.29)
A/A	11	10 (100%)	0 (0%)	11.1 (5.8, 16.7)	1.24 (0.54, 2.84)	1.25 (0.54, 2.86)	33.9 (19.0, 36.2)	1.11 (0.38, 3.20)	1.13 (0.39, 3.27)
P value *			0.16		0.69	0.82		0.96	0.94
G/A or A/A		37 (80%)	9 (20%)	9.2 (6.7, 11.7)	1.28 (0.70, 2.33)	1.21 (0.66, 2.23)	33.9 (23.5, 42.9)	1.12 (0.50, 2.50)	1.00 (0.44, 2.28)
P value *			0.49		0.40	0.53		0.79	1.00
CTLA4 rs231777									
C/C	47	32 (73%)	12 (27%)	9.5 (6.7, 14.5)	1 (reference)	1 (reference)	36.2 (23.2, 42.9)	1 (reference)	1 (reference)
C/T	12	10 (83%)	2 (17%)	10.6 (8.0, 14.7)	1.04 (0.51, 2.09)	1.05 (0.51, 2.15)	30.5 (18.4, 41.4)	0.93 (0.37, 2.28)	1.06 (0.42, 2.71)
P value *			0.71		0.92	0.89		0.87	0.90

* *P* value was based on Fisher's exact test for response, log-rank test for PFS and OS in the univariable analysis (†) and Wald test for PFS and OS in the multivariable Cox regression model (‡) adjusting for ECOG performance status (0 vs 1), and chemo backbone (FOLFOX vs SOX).

Table S5. Immune regulatory SNPs and outcomes in patients treated with first-line FOLFIRI and bevacizumab in FIRE-3 (Control Cohort 1).

	Tumor Response			Progression-Free Survival			Overall Survival		
	N	PR	SD + PD	Median, ms (95% CI)	Univariable HR (95% CI)†	Multivariable HR (95% CI)‡	Median, ms (95% CI)	Univariable HR (95% CI)†	Multivariable HR (95% CI)‡
PDL1 rs2297137									
G/G	159	87 (59%)	60 (41%)	11.3 (9.8, 12.7)	1 (reference)	1 (reference)	24.8 (21.2, 28.1)	1 (reference)	1 (reference)
G/A	106	61 (62%)	37 (38%)	9.6 (8.6, 11.1)	1.14 (0.87, 1.49)	1.13 (0.85, 1.51)	24.2 (20.8, 27.4)	1.13 (0.84, 1.52)	0.98 (0.71, 1.35)
A/A	17	12 (75%)	4 (25%)	9.7 (5.1, 14.7)	1.10 (0.63, 1.91)	1.16 (0.66, 2.06)	14.1 (9.7, 37.0)	1.38 (0.74, 2.57)	1.24 (0.62, 2.48)
P value *			0.48		0.63	0.65		0.48	0.81
C/T or T/T	123	73 (64%)	41 (36%)	9.7 (8.6, 10.5)	1.13 (0.88, 1.46)	1.14 (0.87, 1.49)	23.8 (18.8, 26.9)	1.16 (0.87, 1.54)	1.01 (0.74, 1.37)
P value *			0.45		0.34	0.36		0.30	0.96
CTLA4 rs231777									
C/C	196	104 (58%)	75 (42%)	10.3 (9.6, 11.3)	1 (reference)	1 (reference)	24.8 (21.2, 28.0)	1 (reference)	1 (reference)
C/T [a]	76	48 (68%)	23 (32%)	8.9 (8.0, 12.5)	1.14 (0.86, 1.52)	1.18 (0.88, 1.58)	23.7 (20.6, 27.6)	1.11 (0.81, 1.51)	1.21 (0.88, 1.68)
T/T [a]	3	1 (33%)	2 (67%)						
P value *			0.22		0.35	0.28		0.51	0.24

* *P* value was based on Fisher's exact test for tumor response, log-rank test for PFS and OS in the univariable analysis (†), and Wald test in the multivariable Cox regression model (‡) adjusting for sex, ECOG performance status, primary tumor resection, number of metastatic sites, adjuvant chemotherapy, LDH, RAS and BRAF mutation status. [a] Grouped together to estimate hazard ratios.

Table S6. Immune regulatory SNPs and outcomes in patients receiving first-line FOLFIRI and bevacizumab in TRIBE (Control Cohort 2).

	Tumor Response			Progression-Free Survival			Overall Survival		
	N	**PR**	**SD + PD**	**Median, ms (95% CI)**	**Univariable HR (95% CI) †**	**Multivariable HR (95% CI) ‡**	**Median, ms (95% CI)**	**Univariable HR (95% CI)†**	**Multivariable HR (95% CI)‡**
PDL1 rs2297137									
G/G	146	84 (60%)	57 (40%)	9.7 (8.8, 11.0)	1 (reference)	1 (reference)	23.4 (20.5, 26.4)	1 (reference)	1 (reference)
G/A [a]	61	35 (58%)	25 (42%)	9.5 (8.3, 13.0)	0.85 (0.60, 1.20)	0.86 (0.58, 1.27)	33.9 (25.0, 45.0)	0.63 (0.45, 0.89)	0.65 (0.45, 0.94)
A/A [a]	6	3 (50%)	3 (50%)						
P value *			0.85		0.34	0.44		0.007	0.021
CTLA4 rs231777									
C/C	143	78 (56%)	62 (44%)	10.3 (9.0, 11.1)	1 (reference)	1 (reference)	27.9 (24.7, 31.6)	1 (reference)	1 (reference)
C/T [b]	69	44 (66%)	23 (34%)	9.3 (8.6, 11.0)	0.90 (0.65, 1.24)	0.88 (0.62, 1.24)	20.8 (19.0, 30.9)	1.12 (0.81, 1.54)	1.18 (0.84, 1.65)
T/T [b]	3	1 (50%)	1 (50%)						
P value *			0.38		0.50	0.46		0.48	0.34

* *P* value was based on Fisher's exact test for tumor response, log-rank test for PFS and OS in the univariable analysis (†), and Wald test in the multivariable Cox proportional hazards regression model (‡) adjusting for sex, age, ECOG performance status, primary tumor site, number of metastatic sites, BRAF mutation status, resection of the primary tumors, and adjuvant therapy. [a,b] Grouped together to estimate hazard ratios.

© 2020 by the authors. Licensee MDPI, Basel, Switzerland. This article is an open access article distributed under the terms and conditions of the Creative Commons Attribution (CC BY) license (http://creativecommons.org/licenses/by/4.0/).
